# Scaling effects on arsenic release from excavated hydrothermally altered rocks in column experiments

**DOI:** 10.1007/s11356-023-30594-6

**Published:** 2023-11-15

**Authors:** Xun Du, Toru Inui, Sho Ogata

**Affiliations:** Department of Civil Engineering, Division of Global Architecture, Graduate School of Engineering, Osaka University, 2-1, Yamadaoka, Suita, Osaka, 565-0871 Japan

**Keywords:** Leaching, Hydrothermally altered rock, Arsenic, Column percolation experiment, Scaling effects

## Abstract

The excavation of hydrothermally altered rocks from construction sites in Japan has raised concerns over environmental pollution due to the arsenic (As) release beyond the regulatory limit. An accurate assessment of As leaching from these rocks is imperative to understanding potential environmental implications and formulating efficient containment measures. However, the conduction of column leaching experiments to evaluate As leaching from these rocks encounters a lack of well-established protocols primarily due to the ambiguity surrounding scaling effects resulting from alterations in particle sizes and the corresponding column dimensions. Our study aimed to address this critical issue by conducting column percolation experiments on hydrothermally altered rocks of two distinct particle size ranges and rock layer thicknesses. The pH value was found to be proportional to the specific surface area (SSA) of rocks and the rock layer thickness in terms of H^+^ concentrations. Furthermore, the concentration and leachability of As showed a similar proportionality with the SSA. In contrast, the concentration of As remained relatively unaffected by the increased rock layer thickness, while the leachability of As was noticeably diminished in the column with a thicker rock layer. The absence of elevated As concentration and the decrease in leachability can be attributed to the enhanced As onto Fe/Al oxyhydroxides/oxides within the half-bottom part of the column with a thicker rock layer. Our findings underscore the importance of considering the SSA of rocks and rock layer thickness in the column experiments and help in the design of effective strategies to mitigate environmental contamination.

## Introduction

Arsenic (As), a toxic metalloid, is widely distributed in the earth’s crust. Exposure to this element can induce a multitude of health effects, including cancer, cardiovascular disorders, metabolic disease, and other ailments (Jomova et al. 2011). While it is typically present at a background level of approximately 1 mg/kg, it can become enriched in rocks due to specific geological processes and anomalies, such as hydrothermal alteration (Pirajno 2008).

Hydrothermally altered rocks are prevalent in volcanic areas worldwide, such as Japan. While these rocks typically do not pose any environmental concerns in their natural, anaerobic depositional state, anthropogenic activities such as mining and tunneling expose them to the surrounding ecosystem. If not handled appropriately, these rocks can undergo weathering and oxidization, resulting in the release of toxic elements that can contaminate the soil and groundwater. In search of a proper disposal method and a reuse approach, the leaching behaviors of toxic elements (e.g., arsenic, selenium, and lead) in excavated rocks, including their mechanisms of release and migration, have been widely studied by conducting batch, column, and in situ leaching experiments in recent years (Igarashi et al. 2008; Kamata and Katoh 2019; Kato et al. 2023; Li et al. 2016; Tabelin and Igarashi 2009; Tabelin et al. 2010; Tabelin et al. 2012a, 2012b, 2012c, 2013, 2014a, 2014b, 2017; Tamoto et al. 2015; Tangviroon et al. 2017). Among these experiments, the column experiment has emerged as the preferred method for accurately simulating leaching in a realistic and long-term scenario, as opposed to the conventional batch experiment. In previous studies, rocks have typically been crushed to a particle size of less than 2 mm for column experiments. This size fraction is considered to be the most reactive portion of rocks, and columns with dimensions corresponding to this fraction are relatively easier to set up in laboratory settings. However, in practical disposal and reuse scenarios, excavated rocks can have particle sizes reaching up to 1 m, and rock piles can exceed 10 m in thickness. Despite these practical considerations, studies focusing on the leaching behavior of coarse rocks with larger particle sizes remain scarce. Consequently, further research is necessary in this area to broaden our understanding of the leaching characteristics associated with larger particle–sized rocks and rock layer thickness and to enhance the accuracy of environmental risk assessments.

To investigate the leaching behavior of larger particle–sized rock materials, a column with an increased diameter and height is necessary to ensure sample homogeneity and prevent preferential flow (Inui et al. 2017). However, the impact resulting from changes in particle size and column dimensions on the leaching behavior of As, referred to as the “scaling effects,” is not fully comprehended and necessitates a quantitative evaluation. This evaluation is essential for accurately deciphering laboratory experiment results and predicting in situ leaching behavior. To this end, we conducted column percolation experiments using hydrothermally altered rock specimens with two distinct particle size ranges and two rock layer thicknesses. The pH, redox potential (ORP), and electrical conductivity (EC), as well as the evolution of As and coexisting ions in the effluents, were continually monitored to assess the scaling effects on As leaching.

## Materials and methods

### Materials

The hydrothermally altered rock in this study was collected around an abandoned mining site in Japan. Samples were collected from the ground surface randomly via manual shoveling, and their original size ranged from 20 to 200 mm. Upon transportation to the laboratory, the rocks were air-dried and manually crushed via hammer step by step into five distinct fractions: 19.5–26 mm, 9.5–19.5 mm, 4.75–9.5 mm, 2–4.75 mm, and <2 mm, to ensure the chemical homogeneity across all the fractions. The fraction of <2 mm was partly ground to <50 μm for chemical and mineralogical analyses using an X-ray fluorescence (XRF) spectrometer and X-ray diffractometer (XRD), respectively. Four fractions were reconstituted into 2–9.5 mm (2–4.75 mm × 50 wt.% + 4.75–9.5 mm × 50 wt.%) and 2–26.5 mm (2–4.75 mm ×12.5 wt.% + 4.75–9.5 mm × 12.5 wt.% + 9–19.5 mm ×25 wt.% + ×19.5–26.5 mm × 50 wt.%), both of which were utilized to pack the columns. Additionally, the particle density of rocks was measured as 2.72 kg/m^3^ and As concentration was determined to be 20 μg/L using the Japanese Environment Agency Notification No. 46 test method in 1991.

### Determination of specific surface area

#### Sphericity and specific surface area

In this study, the specific surface area (SSA) of a rock particle was evaluated utilizing the sphericity concept proposed by Wadell (1932). Sphericity $(\overline{{\psi}_{\textrm{s}}})$ is defined as the ratio of the surface area of a sphere with an equivalent volume to the actual surface area of a given particle and expressed mathematically as

1 $${\psi}_{\textrm{s}}=\frac{S_{\textrm{s}}}{S_{\textrm{p}}}=\frac{\pi {d}_{\textrm{v}}^2}{S_{\textrm{p}}}$$

where *S*_s_ is the surface area of a sphere with the same particle volume (m^2^), *S*_p_ is the actual surface area of the particle (m^2^), and *d*_v_ is the diameter of a sphere of the same volume as the particle, calculated by

2 $${d}_{\textrm{v}}={\left(\frac{6m}{\pi \rho}\right)}^{\frac{1}{3}}$$

where *m* is the mass of a particle (kg) and *ρ* is the particle density (kg/m^3^).

SSA (*S*_w_) can be obtained from the mass-surface area relations, the surface area divided by the mass. In conjunction with Eq. (1), SSA can be written as

3 $${S}_{\textrm{w}}=\frac{S_{\textrm{p}}}{m}=\frac{\pi {d}_{\textrm{v}}^2}{\psi_{\textrm{s}}m}=\frac{6}{\rho {\psi}_{\textrm{s}}{d}_{\textrm{v}}}$$

A practical method to evaluate the true sphericity (*ψ*_s_) is using its approximate value, projection sphericity (*ψ*_d_), expressed by Wadell (1935) as

4 $${\psi}_{\textrm{s}}\approx {\psi}_{\textrm{d}}=\frac{d_{\textrm{c}}}{D_{\textrm{c}}}$$

where *d*_c_ is the diameter of a circle of the same area as the projected area of a particle, m, and *D*_c_ is the diameter of a circumscribed circle of a particle projection (m).

Substituting Eq. (4) into Eq. (3), SSA (*S*_w_) can be rewritten as

5 $${S}_{\textrm{w}}=\frac{6{D}_{\textrm{c}}}{\rho {d}_{\textrm{c}}{d}_{\textrm{v}}}$$

#### Average SSA of columns

**Fig. 1:**
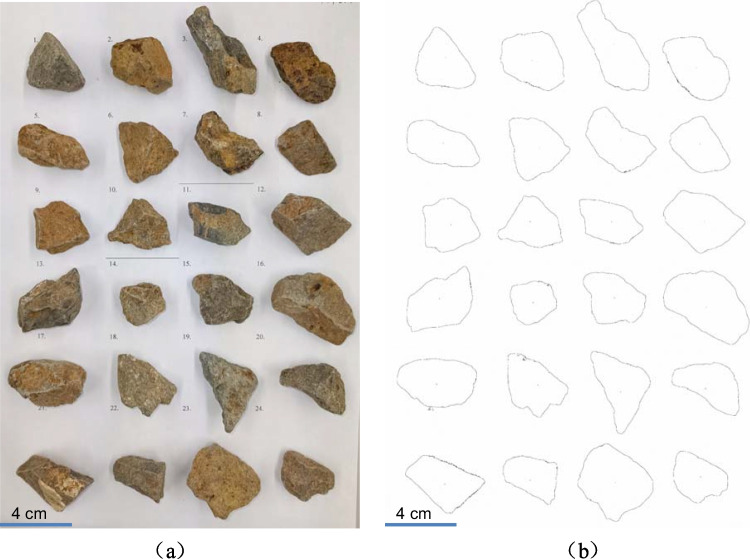
Particle projections used for the analysis of sphericity. **a** The horizontal projection image of particles. **b** The outlines of particles

Two hundred particles were selected randomly from each of the four fractions used in column packing. The mass of each particle was measured and subsequently, the equivalent spherical diameter (*d*_v_) of each particle was calculated by Eq. (2). Following this, the particles were arranged on a white paper with a scale and photographed to obtain a horizontal projection image (Fig. 1a). The image was further processed and analyzed by ImageJ, an open-source image processing software (Schindelin et al. 2012). After the image processing steps such as creating binary images, conversion to black and white, and distinguishing particle boundaries through precise thresholding, the original image was transformed into an image outlining the particles (Fig. 1b). Simultaneously, the circumscribed circle diameter (*D*_c_) and the projected area of each particle were measured in the particle analysis process. Given the projected area of the particle, the area-equivalent diameter (*d*_c_) can be calculated. Finally, the SSA of each particle can be obtained by Eq. (5). The average SSA of each fraction ($\overline{{S}_{\textrm{w}j}}$) can be calculated by the following:

6 $$\overline{{S}_{\textrm{w}j}}=\frac{\sum_{i=1}^{200}\left({s}_{wi}\cdot {m}_i\right)}{\sum_{i=1}^{200}{m}_i}$$

where *s*_w*i*_ is the SSA of a particle (m^2^/kg) and *m*_*i*_ are the mass of a particle (kg).

Ultimately, the SSA of the rocks in a column ($\overline{s_{\textrm{w}}}$) can be calculated by

7 $$\overline{s_{\textrm{w}}}=\sum_{j=1}^4\left(\overline{{s}_{wj}}\bullet {f}_j\right)$$

where *f*_j_ is the mass percentage of a fraction in a column (%).

### Batch leaching experiments

A series of batch leaching experiments were conducted using rock samples of particle size <2 mm and varying liquid-to-solid (L/S) ratios of 1, 2, 5, and 10 mL/g. In each experiment, 200 mL of deionized water was added to a 500-mL polypropylene bottle and mixed thoroughly with rock samples to achieve the desired L/S ratio (e.g., L/S 1 = 200 mL of deionized water/200 g of rock).

The other series of batch leaching experiments were conducted using two particle size distributions, namely, <2 mm and 4.75–9.5 mm. A 100-g rock sample was mixed with 1000-mL deionized water in a-2000 mL polypropylene bottle.

In all batch experiments, the mixture was agitated for 48 h at 200 rpm on a horizontal shaker. Subsequently, the pH and EC of the mixture were measured. The mixture was then filtered through a sterile membrane filter with a pore size of 0.45 μm. The filtrates were acidified and stored at a temperature of 6 °C until chemical analysis was conducted.

### Column experiments

#### Apparatus and initial condition

**Fig. 2:**
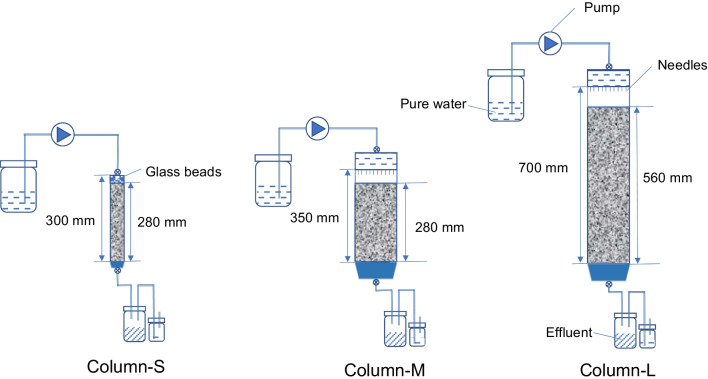
Schematic diagram of the column experimental setup

The column setup and experimental conditions are presented in Fig. 2 and Table 1, respectively. The experimental setup included three cylindrical tubes of different dimensions, namely, *ϕ* 50 mm × *H* 300 mm (Column S), *ϕ* 150 mm × *H* 350 mm (Column M), and *ϕ* 150 mm × *H* 700 mm (Column L). In Column S, a layer of 1-mm-diameter glass beads with a thickness of 20 mm was placed on top of the rock samples to ensure uniform distribution of water. Two rainfall simulators with attached needles were installed at the top of Columns M and L. The rainfall simulator consists of a container with a valve located at the top and 13 needles attached at the bottom. The valve serves to connect this irrigation device to the peristaltic pump, and the needles release water to simulate natural rainfall.

**Table 1:** Experimental conditions of the column experiments

Column name	Particle size (mm)	Inner diameter (mm)	Rock layer thickness (mm)	Bulk density (g/cm^3^)	Porosity	Flow rate (cm/day)
S	2–9.5	50	280	1.55	42.6%	12.2
M	2–26.5	150	280	1.55	42.6%	12.2
L	2–26.5	150	560	1.55	42.6%	12.2

Three columns were built to illuminate the effects of particle size and column thickness. Column S contained particles with a size range of 2–9.5 mm, while Columns M and L contained particles with a size range of 2–26.5 mm. Columns S and M shared a uniform thickness of 280 mm, whereas Column L had twice the thickness of Column M, measuring 560 mm. To ensure consistency, the rock samples were packed to a uniform bulk density of 1.55 g/cm^3^ in all three columns.

#### Irrigation and effluent collection

Deionized water was introduced at a flow rate of 12.2 cm/day in Darcy’s flux through three peristaltic pumps and allowed to flow by gravity. The continuous percolation lasted for 330 days.

Effluents were collected every other day in the sampling bottles at the bottom of each column. After collection, the pH, ORP, and EC of the effluents were measured. Subsequently, the effluents were filtrated through 0.45-μm filters, and the resulting filtrates were acidified and refrigerated for further analysis.

### Post-experimental treatment

After the completion of irrigation, three columns were subjected to blow-drying at room temperature using suction pumps. Column S and Column M were divided into 6 sections, while Column L was divided into 11 sections, as shown in Table 2. Twenty-gram samples (10 g in 2–4.75 mm and 10 g in 4.75–9.5 mm) were randomly collected from sections 1, 3, and 6 in Column S. For Column M, 200-g samples (25g in 2–4.75 mm + 25 g in 4.75–9.5 mm + 50 g in 9–19.5 mm + 100g in 19.5–26.5 mm) were collected from sections 1, 3, and 6. Similarly, for Column L, 200-g samples were collected from sections 1, 3, 5, 9, and 11. All samples were crushed manually by a hammer and then further ground into power by using a pestle and mortar.

**Table 2:** The sections in columns

Column S	Column M	Column L
Section 6 (3 cm thick)	Section 6 (3 cm thick)	Section 11 (6 cm thick)
Section 5 (5 cm thick)	Section 5 (5 cm thick)	Section 10 (5 cm thick)
.	.	.
.	.	.
.	.	.
Section 1 (5 cm thick)	Section 1 (5 cm thick)	Section 1 (5 cm thick)

### Sequential extraction procedure

The current study utilized the sequential extraction procedure, commonly employed for assessing heavy metal contents in soil and sediment, which was originally developed by Tessier et al. (1979) and modified by Marumo et al. (2003). This method originally categorizes As into 5 fractions: exchangeable, carbonates, Fe-Mn oxides, organic, and residual/crystalline. The rocks in this study contain minimal amounts of organic substances. Thus, the organic fraction was not determined in this study. The residual/crystalline fraction, on the other hand, was determined by calculating the difference between the total As content of the sample and the total amount of As extracted in the previous fractions. The methodological parameters and reagents employed for each stage of the process are detailed in Table 3. The exchangeable fraction encompasses As that is both water-soluble and adsorbed specifically. The carbonate fraction, on the other hand, denotes As that is associated with carbonate minerals and can be extracted under mildly acidic conditions. The third fraction is the Fe-Mn oxide fraction, which encapsulates As that is specifically adsorbed or co-precipitated with Fe and Mn oxyhydroxides/oxides. To prevent the loss of solid material, the extractions were performed by mixing 1-g sample with the extractant in polypropylene centrifuge tubes, followed by centrifugation to separate the extract and solid sample. The resultant supernatant was then collected by a syringe and prepared for chemical analysis, while the residue was washed with deionized water. After the second centrifugation, the second supernatant was discarded, and the second residue was utilized in the next stage of the process.

**Table 3:** Sequential extraction for As speciation.

Step	Extractant	pH	L/S ratio (mL/g)	Temperature (°C)	Duration (hour)	Extracted phase
1	1 M NaH_2_PO_4_	5	20	20	1	Exchangeable
2	1 M CH_3_COONa	5	20	20	5	Carbonates
3	0.04 M NH_2_OH·HCl in 25% HOAc	-	20	85	5	Fe-Mn oxides
4		Calculated				Residual/crystalline

### Chemical analysis

Concentrations of Fe, Al, and other cations (Ca, Mg, Mn, etc.) were analyzed by a microwave plasma atomic emission spectroscopy (MP-AES) (4210 MP-AES, Agilent Technologies, Inc. USA). The concentration of As was analyzed by the MP-AES with hydride generation using the multimode sample introduction system (MSIS).

## Results

### Properties of the rock samples

**Fig. 3:**
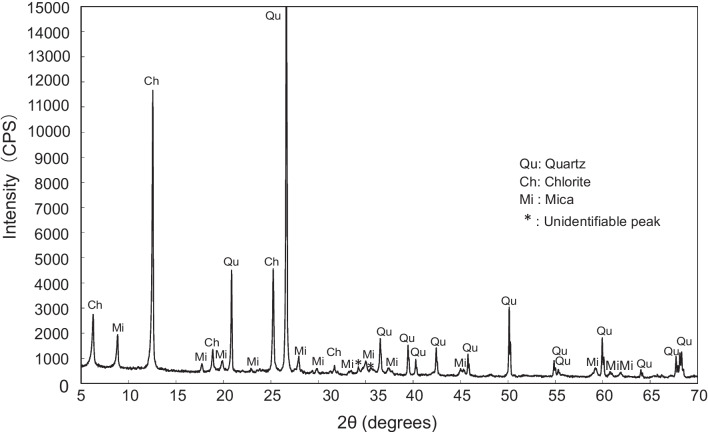
XRD pattern of the hydrothermally altered rock

The chemical and mineral constituents of the hydrothermally altered rock are presented in Table 4 and Fig. 3. Notably, the concentration of As in the rock is exceptionally high, measuring 4015 mg/kg. This value stands in stark contrast to the average As content found in igneous rocks, which typically hovers around 1.5 mg/kg, as reported by Ure and Berrow, (1982). Additionally, the rock exhibits a considerable sulfur (S) content of 1840 mg/kg. However, the XRD analysis did not detect any As-bearing minerals such as pyrite, orpiment (As_2_S_3_), arsenopyrite (FeAsS), or realgar (AsS). The absence of As-bearing minerals suggested that As may be present either as substitutional (with sulfur), as interstitial, or in the adsorbed phase. The principal mineral constituents observed in the rock were quartz, chlorite, and mica. It is essential to acknowledge that chlorite has been reported as a potential source of As by Masuda et al. (2012).

**Table 4:** Chemical composition of the hydrothermally altered rock

Chemicals	Na_2_O	MgO	Al_2_O_3_	SiO_2_	SO_3_	K_2_O	CaO	MnO	Fe_2_O_3_	CuO	As_2_O_3_	PbO
wt.%	0.37	0.55	15.62	59.91	0.46	2.5	0.37	0.22	15.23	0.2	0.53	0.05

### Specific surface area in each column

Table 5 presents a comprehensive summary of SSA for each fraction and column, revealing a notable trend of decreasing SSA with an increase in particle size. Column S exhibits the most extensive SSA of 1.05 m^2^/kg, representing a 2.2-fold increase in SSA when compared to Column M and Column L, both possessing a relatively modest SSA of 0.476 m^2^/kg.

**Table 5:** SSA of each fraction and each column

	Fraction				Column		
	2–4.75 mm	4.75–9.5 mm	9.5-19.5 mm	19.5–26.5 mm	S	M	L
SSA (m^2^/kg)	1.36	0.733	0.360	0.249	1.05	0.476	0.476

### Batch experiments

**Fig. 4:**
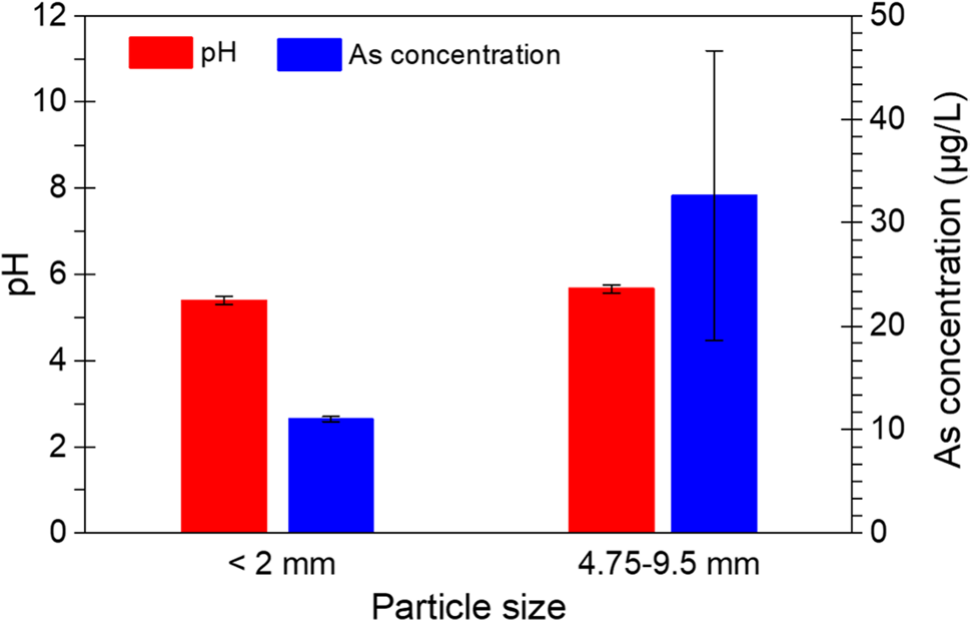
pH and As concentrations in batch experiments using different particle sizes

The pH values and As concentrations in batch experiments, conducted at a L/S ratio of 10 mL/g and utilizing particle size distributions of <2 mm and 4.75–9.5 mm, are depicted in Fig. 4. The pH remained unaffected by the particle size, while larger particles were associated with increased As concentrations.

**Fig. 5:**
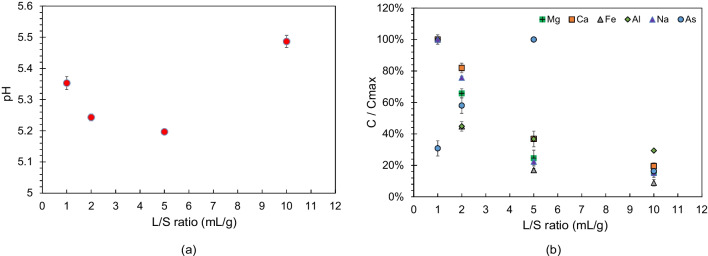
Results in batch experiments using different L/S ratios. **a** pH values at different L/S ratios. **b** Percentages of concentrations relative to the maximum concentration at different L/S ratios

The results of batch experiments using different L/S ratios are presented in Fig. 5. The highest pH value was noted at a L/S ratio of 10 mL/g, while the lowest was observed at a L/S ratio of 5 mL/g. However, the effect of L/S ratios of 1, 2, and 5 mL/g on the pH was deemed insignificant. The greatest concentration of As was observed at a L/S ratio of 5 mL/g, while the greatest concentrations of other cations were found at a L/S ratio of 1 mL/g.

### Column experiments

**Fig. 6:**
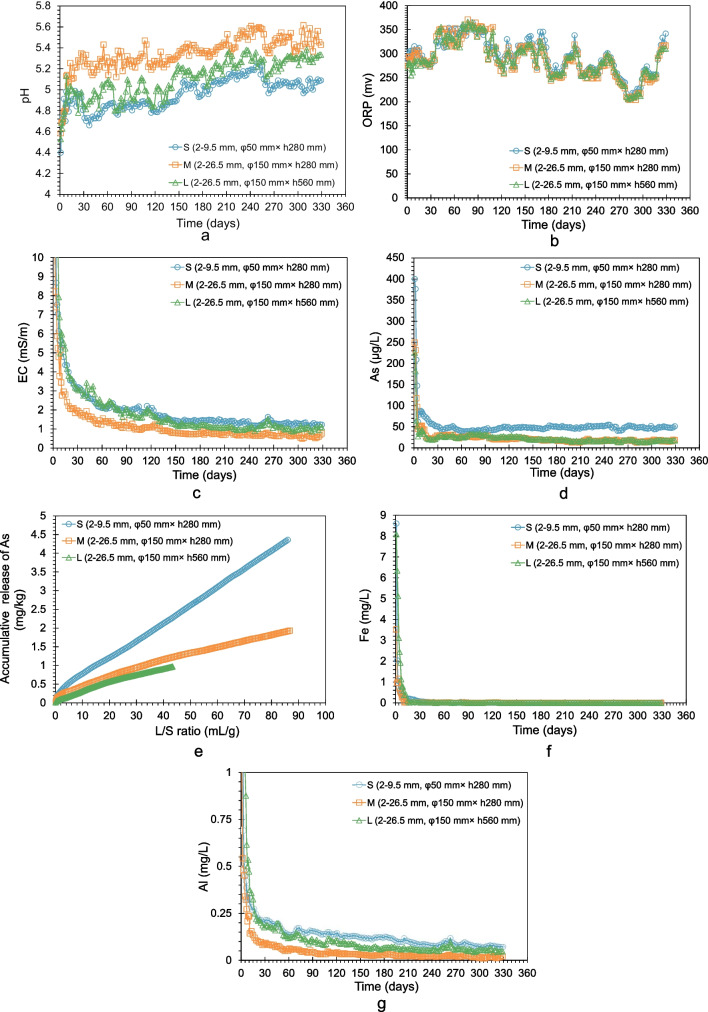
Results of column experiments. **a** Evolution of pH values with time. **b** Evolution of ORP values with time. **c** Evolution of EC values with time. **d** Evolution of As concentration with time. **e** Accumulative release of As with L/S ratio. **f** Evolution of Fe concentration with time. **g** Evolution of Al concentration with time

Fig. 6 compared the results in three columns. The initial pH values in the three columns were the lowest on the first day, after which all pH values increased during the following 20 days before reaching a stable state (Fig. 6a). Subsequently, after 140 days, all pH values started to rise again and eventually reached a stable state around 5.0, 5.4, and 5.2 for Columns S, M, and L, respectively. Throughout the experiment, with the exception of the first 20 days, pH values in Column S were approximately 0.4 pH units lower on average than those in Column M. In comparison, pH values in Column L were on average 0.25 pH units lower. With regard to H^+^ concentration, the concentrations in Columns S and L were 2.5 and 1.8 times higher than those in Column M.

Figure 6b illustrates the significant changes in ORP values over time in three columns, ranging from 228 to 371 mV. Despite these fluctuations, the ORP values in all three columns were consistently comparable and maintained an oxidizing state throughout the entire experimental period. Furthermore, Fig. 6c demonstrates a sharp drop in EC values during the initial 20 days, followed by a gradual decline until stabilization after 140 days. Notably, the EC values in the fine-sized Column S and thick Column L remained persistently higher than those in Column M, with 1.8 and 1.6 times higher on average than those in Column M, respectively.

Evolutions of As concentration with time in the effluents from each column are compared in Fig. 6d. The initial 20 days witnessed a remarkable decrease in As concentration in all three columns. Afterward, the As concentration in Column S exhibited minor fluctuations, ranging between 40 and 55 μg/L. In contrast, the As concentration in Column M and Column L oscillated between 20 and 30 μg/L over the following 120 days before decreasing to nearly 15 μg/L for the remainder of the experiment. Comparing the As concentrations among columns, Column S had 1.5–4 times (with an average of 2.5 times) higher As concentration than Column M, whereas Column L demonstrated nearly identical As concentration with Column S throughout the experimental period.

The L/S ratio, which represents the ratio of the accumulative volume of effluent to the rock mass, was utilized to evaluate the leachability of As. In Column M, the L/S ratio is consistently twice that of Column L. This difference arises from the application of identical irrigation rates, albeit with a halved mass in column M. To enable comparison under the same L/S ratio, the cumulative mass of released As in each column was plotted against the cumulative flow volume expressed in L/S ratio, as shown in Fig. 6e. All three curves exhibited a steady slope after the initial stage, corresponding to relatively constant concentrations. The final As leachability in Column S was 2.2 times that in Column M, whereas the final As leachability in Column L was 0.75 times that of Column M at the same L/S ratio of around 43 mL/g.

Figure 6f and g illustrates the evolution of Fe and Al concentrations in effluents from columns. The concentration of Fe in all three columns showed a rapid decline, plummeting below 0.01 mg/L after 50 days. The Al concentrations, on the other hand, exhibited an abrupt drop during the initial 30 days, followed by a gradual decrease until the conclusion of the experimental period. The average concentration of Al in Columns S and L was 3.6 and 2.6 times higher, respectively, than that in Column M.

### Sequential extraction of As

**Fig. 7:**
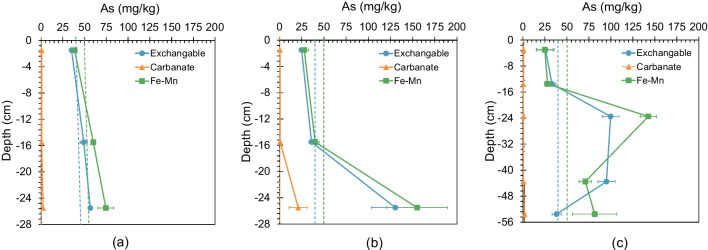
Changes in the As contents of fractions with depth in columns (dashed lines represent the original As content of each fraction). **a** As distribution in Column S. **b** As distribution in Column M. **c** As distribution in Column L

Fig. 7 illustrates the variations in As contents of three As-bearing fractions with depth in three columns and compares these fractions to the As contents in the original rock. The As content in the carbonate fraction was insignificant in all columns and depths, except for the lower half region of Column M. Conversely, As levels in the exchangeable and Fe-Mn fractions increased with depth in Column S, Column M, and the upper half region of Column L. Intriguingly, As concentrations within these two fractions were comparatively lower than the initial levels at a depth of around 3 cm in all three columns, although they approached the original values at a depth of around 15 cm and surpassed the original concentration at a depth of around 24 cm. In the lower half region of Column L, As content in the exchangeable fraction marginally declined with depth, eventually reaching the original value at the base. Meanwhile, As content within the Fe-Mn fraction in the lower part of Column L declined relative to the middle part, although it remained higher than the original content.

## Discussion

### Source of As in the hydrothermally altered rock sample

Sulfide minerals and chlorite are two potential sources of As in the hydrothermally altered rocks according to the XDF and XRD results. The chemical property of As is similar to that of S, enabling the former to substitute for the latter in crystal structures of many sulfide minerals, like galena (PbS) and chalcocite (Cu_2_S) (Smedley and Kinniburgh 2002). As could also substitute for silicon (Si) in tetrahedral and octahedral sites in silicate minerals, like chlorite (Masuda et al. 2012). The majority of As content, approximately 98%, is found in the residual/crystalline fraction, predominantly comprising silicates and sulfides. This finding confirms that a substantial portion of As is integrated into the mineral structures in the “Sequential extraction of As” section. Consequently, As bound within these structures can only be released through the oxidation of sulfides or chemical weathering of chlorite.

**Fig. 8:**
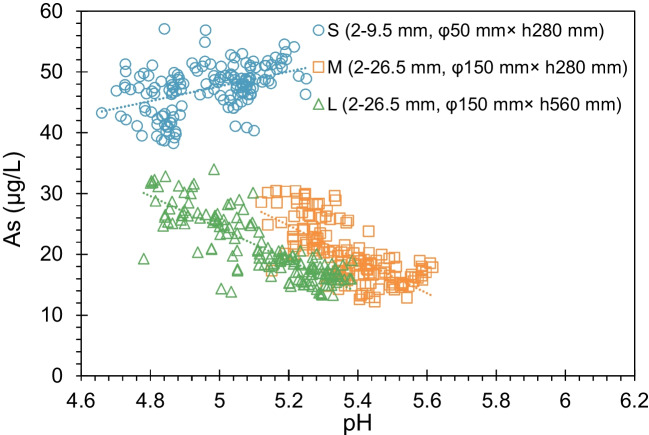
Correlation between As concentrations and pH values

One of the characteristics of the oxidation of sulfide minerals is the acid pH values in effluents. Concentrations of As in the effluent of three columns with respect to the pH values are plotted in Fig. 8. Data within the initial 20 days were excluded due to the dominant influence of weathering products during the rock’s exposure before collection, significantly impacting the leaching process. The consistently acidic pH values in the three columns throughout the experiment duration suggested an ongoing sulfide mineral oxidation. The correlation between pH values and As concentrations in Columns M and L further supported the association of As leaching with sulfide oxidation. However, this correlation was absent in Column S. Interestingly, the increase in pH values after 140 days had minimal effect on the As concentration in Column S (Fig. 6a, d). This finding indicated that the oxidation of sulfides might not solely account for As release. Additional research by Masuda et al. (2012) has reported that the chemical weathering of chlorite can also lead to As release, forming nanocrystalline goethite (FeO(OH)), with the mobilization of As subsequently influenced by the adsorption of Fe-oxyhydroxides. The As content in the Fe-Mn fraction through sequential extraction procedure provides supporting evidence for the existence of Fe-oxyhydroxides. Based on these results, both sulfide minerals and chlorite could be potential sources of As, with the decomposition of sulfide minerals and chlorite serving as the primary mechanisms for As release.

### Particle size effect

Numerous studies have demonstrated a linear correlation between the oxidative dissolution rate of As-bearing sulfides and chlorites and the SSA (Liao et al. 2021; Moses and Herman 1991; Nicholson et al. 1988; Pugh et al. 1984). Assuming a homogeneous distribution of As sources in hydrothermally altered rocks, the amount of As generated during oxidative dissolutions should be proportional to the SSA of rock samples. However, SSA also influences other factors that can impact the mobilization of As, including the release of iron and change in pH values. Therefore, the effect of particle size on As release is a combined outcome of the dissolution rate and porewater chemical composition.

The effect of particle size on the behavior of As in batch and column experiments has been investigated in this study. The effect of particle size in batch experiments differed from that observed in column experiments. The primary reason for this difference can be attributed to chemical equilibrium. Batch experiments, having sufficient time to reach chemical equilibrium, allowed processes such as precipitation/coprecipitation and adsorption to effectively restrict the migration of As. Smaller particle size increased the reactive surface area and thereby increased the dissolved As concentration. On the other hand, high cation release (e.g., Fe and Al) led to extensive adsorption of As, resulting in a lower final concentration of As in batch experiments. Conversely, all column experiments in this study did not achieve chemical equilibrium due to the rapid renewal of pore water. This non-equilibrium state could be evidenced by lower pH values in column experiments compared to batch experiments and higher EC values in Columns S and L compared to Column M. The retardation of As migration in non-chemically equilibrium environments was found to be less significant than in batch experiments. Therefore, in column experiments, the effect of increasing surface area was particularly significant, with As leaching found to be proportional to the specific surface area.

The pH of effluents derived from hydrothermally altered rocks is primarily governed by the dissolution of carbonate minerals, sulfide oxidation, and precipitation and hydrolysis reactions (Tabelin et al. 2012a). However, in our study, mineral composition analysis in “Properties of the rock samples” section revealed the absence of carbonate minerals, such as calcite. Additionally, the Ca^2+^ concentration was less than 3 mg/L in this study, while Tabelin et al. (2012a) reported Ca^2+^ concentrations exceeding 50 mg/L in their column experiment. Therefore, the dissolution of carbonate minerals did not play a significant role in controlling the pH in our study. Instead, the acidic pH of the effluent could be mainly attributed to the oxidation of sulfide minerals. The elevated release of Fe and the low pH value observed in the early stages of the column experiment are attributed to the dissolution of soluble secondary products resulting from the dissolution of sulfide minerals and chlorite due to long-time exposure of rocks. As the experiment progressed, the pH increased and Fe oxides/oxyhydroxides did not dissolve under the pH value within the columns, as evidenced by the near-zero Fe concentrations in the rest of the experiment.

Tamoto et al. (2015) performed column experiments on excavated sedimentary rocks under in situ conditions, employing two distinct particle sizes, namely, particles less than 2 mm and those between 2 and 9.5 mm. Unlike the current investigation, they reported that the particle size exerted negligible influence on the leachability of As and did not yield a consistent and stable variation in effluent pH values. Their research demonstrated that the exchangeable fraction of As accounted for approximately 34% of the total As content and the dissolution of As-bearing soluble salt emerged as a crucial release mechanism. Thus, it appears that the magnitude of the particle effect is closely linked to the release mechanism of As and the forms of As present in the rocks. Furthermore, the difference in irrigation method employed in the experiments may have contributed to the observed variances. Intermittent natural rainfall was the only influent in their in situ experiments. Following rain, pore water between the rock particles remained partially preserved for a certain period. This allowed for a more extensive period to achieve chemical equilibrium which resulted in similar As releases and pH values in effluents.

### As adsorption onto Fe/Al oxides/oxyhydroxides

Previous research has highlighted the crucial role of Fe/Al oxides/oxyhydroxides in immobilizing As (Dousova et al. 2003; Giles et al. 2011; Wang and Mulligan 2006). In our study, this immobilization effect was also observed in our batch and column experiments.

Batch experiments at various L/S ratios indicated that, except for As, chemical concentrations tended to decrease as L/S increased, owing to a reduction in sample dilution with leachant. The concentrations of As were significantly lower at L/S ratios of 1 and 2 mL/g, suggesting that As release is largely influenced by immobilization processes. A higher Fe/As ratio was found to result in lower final As concentration in batch experiments, primarily due to extensive precipitation of Fe oxides/oxyhydroxides (Jia and Demopoulos 2005). Accordingly, the present study showed that the high Fe and Al concentrations at low L/S ratios led to low As concentration, mainly as a result of enhanced Fe/Al oxides/oxyhydroxides precipitation and adsorption.

The sequential extraction procedure analysis demonstrated that As contents in exchangeable fraction and Fe-Mn fraction in the region with a depth of > 24 cm were higher than those in the original rock samples. High Al concentration in the extract from Fe-Mn fraction suggested that As associated with Al oxides/oxyhydroxides was also released in this extraction step. Thus, it can be inferred that As adsorption onto Fe/Al oxides/oxyhydroxides was more significant in the region with a depth of > 24 cm. This outcome is in agreement with previous research by Tabelin et al. (2012a), which noted that the top half of the 20 cm column was the site of maximum As leaching, while the lower half was characterized by adsorption. The increased adsorption of As in the lower section of the columns may be attributed to two potential reasons. Firstly, the oxidation of As(III) to As(V) may occur as it flows downwards within the column, leading to the gradual dominance of As(V) in the pore water due to longer reaction times (Tabelin et al. 2012c). The preferential pH range for the adsorption of As(V) onto ferrihydrite is reported to be within 3.8–7.4, whereas that for As(III) is within 8.2–10 (Raven et al. 1998). In the present experiments, As(V) was readily adsorbed within the pH range of 4.8–5.4. Thus, the shift in As speciation and pH-dependent adsorption ultimately resulted in increased As adsorption. Secondly, the elevation of As concentration with depth may also contribute to the increased As adsorption in the lower section of the column. Prior research has reported that elevated As concentration could increase the amount of adsorbed As onto Fe/Al oxides/oxyhydroxides (Youngran et al. 2007). When the As concentration increased with depth, As immobilized by Fe/Al oxides/oxyhydroxides increased.

**Fig. 9:**
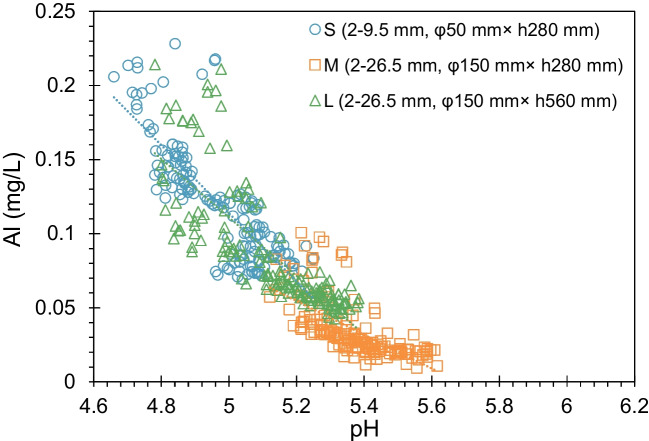
Correlation between Al concentrations and pH values

The As contents in exchangeable and Fe-Mn fraction at a depth of around 24 cm coincided well in Columns M and L but were much higher than those in Column S. Moreover, As content in these two fractions in the bottom half part of Column L decreased with depth. These observations could be attributed to the dissolution of Al oxides/oxyhydroxide under acid conditions. The lower pH in the bottom part of Column S and the bottom half part of Column L facilized the dissolution of Al oxides/oxyhydroxide, resulting in the relatively lower As content in exchangeable and Fe-Mn fractions. The pH effect on the dissolution of Al oxides/oxyhydroxide could be supported by the correlation between Al concentrations and pH values in Fig. 9.

### Rock layer thickness effect

The rock layer thickness effect is primarily characterized by the comparable As concentrations in Columns M and L and the lower leachability in Column L. The concentration of H^+^ in the effluent of Column L was twice that in Column M, which suggested that As amount generated from sulfide oxidation in Column L was also twice that in Column M. The extensive adsorption of As onto Fe/Al oxides/oxyhydroxides in the bottom part of Column L resulted in similar As concentrations in the effluent to those in Column M.

A similar rock layer thickness effect was also observed in previous studies. For instance, Tabelin et al. (2012a) reported that As concentrations in 200 mm and 250 mm columns were comparable but lower than those in the 100 mm column in their column experiments using hydrothermally altered rock of less than 2 mm. Tamoto et al. (2015) conducted another column experiment using sedimentary rock of less than 2 mm and found that As concentration in the 300 mm column was slightly lower than that in the 600 mm column, but the leachability in the former was still higher than that in the latter. Both studies primarily attributed lower leachability in increased thickness of the rock layer to enhanced adsorption of As onto Fe/Al oxides/oxyhydroxides. They further explained that the saturation of Fe and Al was more likely to reach in the bottom part of the column as pore water flowed down the column, resulting in the precipitation of Fe/Al oxides/oxyhydroxides. Although our experiments differed from the previous studies in rock source, particle size, leaching method, column size, and pH range and composition of the effluent, we also observed an increase in As adsorption by Fe/Al oxides/oxyhydroxides due to thicker rock layer. Both Fe and Al are widely present in rocks, particularly in As-bearing rocks, where both elements are secondary products of rock weathering, resulting in their high correlation in leaching. Fe and Al readily precipitate to form Fe/Al oxides/oxyhydroxides over a wide range of pH values. For instance, Fe^[3+]^ can precipitate at pH >3.5 and Al^[3+]^ can precipitate at pH of 5–9 (Wei et al. 2005). The longer water contact time due to a larger rock layer thickness can affect the As speciation and concentration, as mentioned in the “As adsorption onto Fe/Al oxides/oxyhydroxides” section, and facilitate the oxidation of Fe^[2+]^ to Fe^[3+]^. Fe^[2+]^ is reported to be precipitated at pH>8 (Wei et al. 2005); thus, the oxidation of Fe^[2+]^ to Fe^[3+]^ can result in more extensive precipitation of Fe oxides/oxyhydroxides.

### Engineering implications

The dimensions of excavated rocks in disposal or utilization scenarios significantly surpass those in laboratory experiments. This study examines the scaling effect resulting from variations in particle size and rock layer thickness on the release of As from hydrothermally altered rocks under column conditions. Understanding this effect can facilitate the explanation of As leaching discrepancies between laboratory experiments and actual disposal, leading to appropriate disposal or reuse techniques to minimize As release in practice.

Increasing the particle size of hydrothermally altered rock can decrease the concentration and total release of As, as well as the system’s acidity. Furthermore, the quantitative relationship between SSA and As leaching can be an effective method for predicting actual leaching based on laboratory results. Tabelin et al. (2012a) discovered that the finer particle size in column experiments resulted in higher As leaching than that observed in actual waste rocks and proposed that laboratory results could be a conservative estimate of As leaching. Column experiments aim to provide accurate leaching assessments to avoid over- or underestimations, as this evaluation is crucial for waste treatment. Overly conservative estimates may result in the wastage of recyclable waste and increased disposal expenses. The combination of laboratory results and SSA differences between laboratory and field samples could provide a realistic prediction of actual leaching, helping to design a cost-effective disposal method.

The greater rock layer thickness may enhance As adsorption on Fe/Al oxides/oxyhydroxides, thus decreasing As leachability. Increasing rock layer thickness during actual disposal could reduce total As release, but actual rock layer thickness is often one to two orders of magnitude larger than those observed in laboratory experiments. The significant alterations in coexisting ion concentration and pH value by such a large thickness may have a considerable impact on As leaching. Furthermore, several studies have indicated that greater thickness could lead to a slightly higher As concentration, as previously mentioned, although our study observed no such influence on As concentration. Therefore, increasing thickness to reduce As leaching in practice requires considerable caution, and further investigations into the influence of thicker rock layers, particularly those much closer to actual thickness, are recommended.

## Conclusions

The scaling effects on non-anthropogenic As leaching from the hydrothermally altered rock, triggered by particle size and thickness, were investigated by performing column experiments with two particle size ranges and two rock layer  thicknesses. The main findings of this study are summarized as follows:

1. Both larger SSA of rocks and rock layer thickness led to lower effluent pH values and the concentration of H^+^ corresponding to the pH value was proportional to the SSA of rocks and the rock layer thickness of the column.
2. As concentration and leachability with respect to the L/S ratio were virtually proportional to the SSA of rocks.
3. Greater rock layer thickness did not increase As concentration and even decreased the leachability with respect to the L/S ratio due to the enhanced As adsorption onto Fe/Al oxyhydroxides/oxides.

These findings could contribute to the interpretation of laboratory column experiment results, the relationship between the laboratory results and the actual release under field conditions, and the design of an alternative disposal method for hydrothermally altered rocks. Future work will focus on a numerical model that takes the scaling effects into account.

## Data Availability

Not applicable.
